# Caesarean Delivery Complicated by Unintentional Subdural Block and Conversion Disorder

**DOI:** 10.1155/2013/751648

**Published:** 2013-11-14

**Authors:** Hesham Elsharkawy, Ashish K. Khanna, Sabri Barsoum

**Affiliations:** ^1^Anesthesiology Institute and Outcomes Research, Cleveland Clinic Foundation, 9500, Euclid Avenue, Cleveland, Ohio, OH 44195, USA; ^2^Cleveland Clinic Learner College of Medicine of Case Western Reserve University, Cleveland, Ohio, OH 44195, USA; ^3^Anesthesiology Institute, Cleveland Clinic Foundation, 9500, Euclid Avenue, Cleveland, Ohio, OH 44195, USA; ^4^Division of Obstetric Anesthesiology, Hillcrest Hospital, Cleveland Clinic Foundation, 6780, Mayfield Road, Mayfield Heights, Ohio, OH 44124, USA

## Abstract

Combined spinal epidural (CSE) can provide excellent labor analgesia. Subdural block is also a potential but rare complication of attempted epidural placement during a CSE procedure, which may present as a block that is usually patchy in nature, with a component of sensory and/or motor deficit and a variable duration of action. In addition, a conversion disorder or a functional neurological disorder has been described with epidural and spinal anesthesia in obstetric patients. In this clinical report, we describe a 33-year-old G4P3 at 40 weeks gestation that received an unintentional subdural block as part of her labor analgesia and after an uneventful caesarean delivery presented with a conversion disorder. The rarity of the association between a subdural block and a conversion disorder complicated by the fact that the neurological deficit produced by the subdural block and that produced by a conversion disorder are similar in distribution made the clinical presentation and diagnosis a challenge for the obstetric anesthesia team. A functional neurological disorder of this nature complicating a subdural block in an obstetric anesthesia clinical practice has not been described so far.

## 1. Introduction

Combined spinal epidural (CSE) can provide excellent labor analgesia. Complications of this procedure are uncommon, but they may include bleeding, infection, failed block, and nerve root injury [1]. Subdural block is also a potential but rare complication of attempted epidural placement during a CSE procedure, which may present as a block that is usually patchy in nature, with a component of sensory and/or motor deficit and a variable duration of action. In addition, the literature is rampant with reports of a conversion disorder or a functional neurological disorder in conjunction with epidural and spinal anesthesia in obstetric patients [2, 3]. However, this type of conversion disorder complicating a subdural block has not been described so far. In this report, we describe a patient who had a conversion disorder after undergoing a caesarean delivery following an unintentional subdural injection. The rarity of the association between a subdural block and a conversion disorder complicated by the fact that the neurological deficit produced by the subdural block and that produced by a conversion disorder are similar in distribution made the clinical presentation and diagnosis a challenge for the obstetric anesthesia team.

## 2. Case Description

A 33-year-old G4P3 at 40 weeks gestation presented to the labor and delivery unit in active labor. Her past medical history was significant for postpartum depression and psychosis during each of her previous three pregnancies. On admission, her vital signs were within normal limits and the fetal heart showed good beat-to-beat variability with a rate in the 120′s. In the past, the patient had received epidural anesthesia for her prior vaginal deliveries without any complications. Shortly after admission, the patient requested labor analgesia, for which a CSE was set up. This was achieved in the sitting position with a sterile technique at the L4-5 interspace using an 18-gauge Tuohy needle. Loss of resistance to saline was obtained easily. A 25-gauge Whitacre spinal needle was introduced through the Tuohy needle, and cerebrospinal fluid was appreciated. Further, 1.2 cc of isobaric bupivacaine 0.25% and 25 micrograms (mcg) of fentanyl were given via the spinal needle. Subsequently, the needle was removed, and the epidural catheter was placed easily through the Tuohy needle already in-situ. Aspiration was negative for CSF and/or blood. Baseline blood pressure and heart rate were 110/62 mmHg and 91/min, respectively. An epidural test dose with 3 mL lidocaine 1.5% with 1 : 200,000 epinephrine was negative. She was then placed supine and observed for 10 minutes, during which vitals were within 10% of baseline. Twenty-five minutes later, fetal decelerations to 70 and maternal hypotension to the order of 80/40 mmHg occurred. One litre lactated ringer, phenylephrine 100 mcg, and ephedrine 10 mg were administered intravenously. Blood pressure responded transiently, but she could not sustain hemodynamic stability. On sensory examination at this stage, she was noted to have a T10 level of blockade. Persistent fetal decelerations were evident, and she was prepped for caesarean delivery in the operating room. The epidural catheter was bloused with 5 mL of 0.5% isobaric bupivacaine. Initially, the block level remained at T10. However, a repeat exam after 15 minutes demonstrated a block level at T3, she still retained sensation in the lower extremities. At this stage, reaspiration of the catheter revealed some CSF. This was not a free flow and stopped on repeated attempts at reaspiration. All this time the patient was hemodynamically stable and had no signs of respiratory depression. The intraoperative course was unremarkable, with fetal delivery six minutes after skin incision. APGAR scores at one and five minutes were eight and nine, respectively. The patient was taken to the recovery unit, at which time she was arousable, able to breathe without difficulty, and had stable vital* *signs. However, generalized weakness and difficulty moving upper extremities were noted. Per institutional protocol, at this stage the epidural catheter was removed with its tip intact. Concurrently done coagulation studies were normal. She was observed in recovery for six hours, without significant improvement. Neurology was then consulted for further workup. Upon examination, the neurologist observed diversion of strength of the forearms with a grip that had a somatic component. The patient's strength increased in either hand when she was asked to release the grip in the opposite hand. An MRI scan of the spine was completely normal. The neurological signs and symptoms were not consistent with the examination findings, and no objective neurologic abnormality could be found. This unexplained sensory and motor weakness of the upper extremity persisted till postoperative day 3, following which she had a spontaneous recovery that was fully functional and exhibited no motor or sensory deficit. She was ultimately diagnosed with a conversion disorder as a diagnosis of exclusion. She was discharged on postoperative day four, with no further neurological deficits and a stable neurological exam.

## 3. Discussion

Subdural injection is a rare though not unknown complication of spinal, epidural, and a combined spinal-epidural (CSE) block. Interestingly, obstetric patients receiving these modalities of analgesia for labor have been cited in the literature to have the highest incidence of this complication [4]. Jenkins et al., in one such study of greater than 100,000 obstetric epidurals found the incidence of subdural injection to be 0.024% [5]. Anatomically, the subdural space is a narrow potential space between the arachnoid and the dura mater. It extends cranially from the cranial cavity to caudally up to the lower border of the second sacral vertebra [6, 7]. A subdural block is most commonly present after an epidural bolus injection, though presentations with a spinal and a CSE have also been described. The typical neurological block is very variable with both a minimal patchy and also a widespread dermatomal spread as possible outcomes. The block invariably does not correspond to the dose and volume of the local anesthetic injected in the epidural space. Onset is slow and a high block usually manifests as a respiratory discoordination rather than a respiratory arrest. Recovery time may be as much as six hours following injection [1, 8]. In our case, the patient displayed a high patchy block that had a delayed time of onset. Our patient had a dense block from T3 down to the high lumbar dermatomes. However the lower lumbar dermatomes appeared to be spared. The cerebrospinal fluid (CSF) aspiration was positive for about 1 cc volume but later on was negative again. This did raise the suspicion for a subarachnoid migration of the catheter. However, the CSF aspirated was not free-flowing and stopped afterwards. This would be explained by the rent in the dura created by the spinal needle that allowed some CSF to leak into the subdural space. Back leak of CSF would have also distended this subdural space further, which would have aided the epidural catheter placement into this space [4]. Despite the high dermatomal level the patient's hemodynamic and respiratory status were stable. The intraoperative course was unremarkable, and the patient and baby were stable in the recovery unit. Subsequently we followed our institutional protocol and removed the catheter, since we were uncertain of its correct position in the face of unexpected neurological findings in this patient. It is important to note that no clear-cut guidelines exist in the literature about the management of the epidural catheter in the subdural space [9]. However, our patient continued to have upper extremity weakness with both a motor and sensory component. This was unusual considering that it was more than six hours since the last dosing of the epidural, which had now been removed. The clinical neurological examination did not at any stage corroborate with the patients subjective symptoms of sensory and motor deficit in the upper extremities. Moreover, her motor weakness appeared to decrease when she was made to focus on other motor tasks (such as concentrate on the motor weakness of her other arm). We kept a provisional diagnosis of a conversion disorder, and a clean imaging study of the spine further confirmed this. Conversion disorder (CD) is a type of somatoform disorder in which there is dysfunction of a voluntary sensory or motor activity, without objective neurologic findings. It is thought to be multifactorial in etiology, and the somatic complaints and symptoms invariably represent an underlying emotional distress [2]. CD may coexist with major depressive, personality, or generalized anxiety disorders. The American Psychiatric Association (APA) in the latest diagnostic and statistical manual (DSM-V) has proposed to describe this disorder under the broad heading of Functional Neurological Disorders [10].

The section below shows the precise diagnostic criteria for a conversion disorder as listed by the American Psychiatric Association (APA).

*Criteria for Diagnosis of Conversion Disorder.* Criteria A, B, C, and D must all be fulfilled to make the diagnosis.

- One or more neurologic symptoms such as altered voluntary motor, sensory function, or seizure-like episodes.
- The symptom, after appropriate medical assessment, is not found to be due to a general medical condition, the direct effects of a substance, or a culturally sanctioned behavior.
- The physical signs or diagnostic findings are internally inconsistent or incongruent with recognized neurological disorder. * *
- The symptom causes clinically significant distress or impairment in social, occupational, or other important areas of functioning or warrants medical evaluation.

Adapted from American Psychiatric Association (APA) Diagnostic and Statistical Manual (DSM)-V (Proposed revision of DSM-IV).

The section below highlights the clinical manifestations of a subdural block. It can be easily understood that these two clinical entities can be present in a very similar manner.

*Any or All of the Criteria A–D May Be Consistent with a Diagnosis of Subdural Block*

- Delayed onset after an attempted neuraxial block;
- variable neurological distribution not consistent with drug dosage and concentration;
- minimal sympathetic blockade;
- usually no respiratory arrest.

Obstetric patients who have received a spinal, an epidural, or a CSE for labor analgesia and or operative delivery have been commonly described to have conversion disorders [2, 5].

However, this has not been described with an unintentional subdural block as in our patient. The fact that the subdural block presents with variable levels of sensory and/or motor block with a prolonged duration of action further complicated our diagnoses. Our patient initially had a delayed onset patchy block because of subdural injection. This explained the distribution of the blockade in an area limited to the upper thoracic and the upper lumbar dermatomes. This led us to believe that our patient had a prolonged duration of block in the recovery room due to this initial subdural injection. However, in the recovery room there was no evidence of any neurological blockade except her upper extremity weakness and coexisting sensory deficit. In addition, it would be difficult for a subdural block to persist greater than six to ten hours after the initial injection, considering the dosage of local anesthetic we had used. In addition, a subdural block does not manifest as a singular upper extremity weakness and or sensory deficit that persists in isolation [9]. These observations, prompted us to think above and beyond a subdural block and in close consultation with neurology we arrived at a diagnosis of a conversion disorder.

Treatment involves informing the patients that they have a somatoform condition and approaching them in an empathetic rather than confrontational manner. Exhaustive medical tests and pharmacotherapy should be minimized [10]. Approximately twenty-four hours postoperatively, our patient still complained of generalized weakness. After a diagnosis of conversion disorder was established, we used the above treatment strategies to alleviate her symptoms.

Any doubt that this was a conversion disorder was further confirmed by normal spine imaging and also that the patient had persistent weakness till postoperative day 3 which resolved spontaneously without any lasting deficit.

## 4. Conclusion

A subdural injection is a rare complication of spinal, epidural, or a CSE used for labor analgesia. Conversion disorder can complicate anesthetic management of the parturient but can be easy to be diagnosed in case of a spinal or epidural anesthetic. However, the neurological signs of conversion can substantially overlap those of an unintentional subdural block, in that both have variable levels and durations of the blockade. Indeed, this entity has not been described in association with a subdural block. The obstetric anesthesiologist should be aware that an excessively prolonged subdural block with an inconsistent neurologic examination could be a conversion disorder. This also demands working in close conjunction with the neurologist and neuropsychiatrist in the obstetric suite.
